# Supplementary figures and images for: IoT-IIRS: Internet of Things based intelligent-irrigation recommendation system using machine learning approach for efficient water usage
Source: PeerJ Comput Sci. 2021 Jun 21;7:e578. doi: 10.7717/peerj-cs.578 (PMC8237332; doi:10.7717/peerj-cs.578)

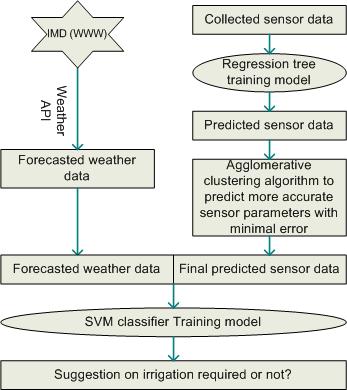

Supplement: Supplemental Information 1 — All the ML models have implemented using the Scikit-learn Python library. To validate the effectiveness of the system NIT Raipur and our own collected data have been used. [file peerj-cs-07-578-s001.zip › code_dataset/cs-54857-ML_model.jpg]

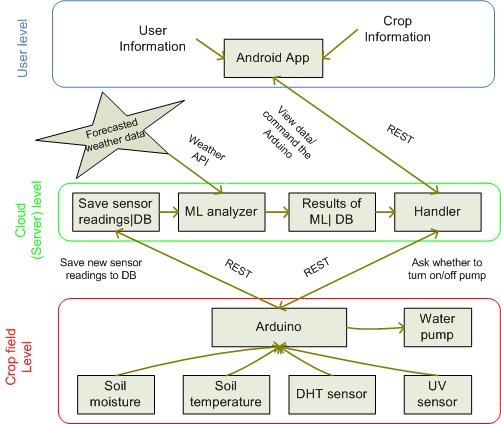

Supplement: Supplemental Information 1 — All the ML models have implemented using the Scikit-learn Python library. To validate the effectiveness of the system NIT Raipur and our own collected data have been used. [file peerj-cs-07-578-s001.zip › code_dataset/cs-54857-Smart_Irrigation.jpg]
